# Investigation of Gallium Arsenide Deformation Anisotropy during Nanopolishing via Molecular Dynamics Simulation

**DOI:** 10.3390/mi15010110

**Published:** 2024-01-08

**Authors:** Bo Zhao, Xifeng Gao, Jiansheng Pan, Huan Liu, Pengyue Zhao

**Affiliations:** 1 Center of Ultra-Precision Optoelectronic Instrumentation Engineering, Harbin Institute of Technology, Harbin 150001, China; 22b901011@stu.edu.cn (B.Z.); 22b301002@stu.edu.cn (J.P.); liuhuanxues@163.com (H.L.); pyzhao@hit.edu.cn (P.Z.); 2 Key Lab of Ultra-Precision Intelligent Instrumentation, Ministry of Industry Information Technology, Harbin 150080, China

**Keywords:** molecular dynamics, crystal orientation, nanopolishing, surface quality

## Abstract

Crystal orientation significantly influences deformation during nanopolishing due to crystal anisotropy. In this work, molecular dynamics (MD) simulations were employed to examine the process of surface generation and subsurface damage. We conducted analyses of surface morphology, mechanical response, and amorphization in various crystal orientations to elucidate the impact of crystal orientation on deformation and amorphization severity. Additionally, we investigated the concentration of residual stress and temperature. This work unveils the underlying deformation mechanism and enhances our comprehension of the anisotropic deformation in gallium arsenide during the nanogrinding process.

## 1. Introduction

Gallium arsenide, as a III-V compound semiconductor, exhibits direct bandgap characteristics when compared to traditional elemental semiconductor materials such as silicon (Si). It finds extensive applications in the manufacturing of laser diodes [1] and offers reduced noise levels in high-frequency operating conditions compared to silicon devices [2]. Furthermore, gallium arsenide material demonstrates high carrier mobility [3] and optical coupling effects [4], making it well-suited for next-generation communication and advanced optical device fabrication [5,6]. However, during semiconductor processing, surface defects induced by fabrication processes have a significant impact on the electrical characteristics and service life of the final devices [7]. Existing research has indicated that the crystal orientation of gallium arsenide surfaces significantly influences the performance of the final processed devices, and selecting different crystal orientations during processing can result in substantial enhancements of semiconductor components [8,9].

Due to the significant impact of surface defects generated during semiconductor device processing on the final quality, scholars have conducted extensive experimental research. These experiments primarily include indentation tests and scratch tests [10,11], which employ experimental methods to observe structural surface defects and subsequently investigate surface morphology and crystal structure damage. Gao et al. [12] utilized molecular dynamics to examine GaAs laser bar cleavage. Their study highlighted the influence of scratching depth on scratch quality and provided optimal parameters for GaAs cleavage. Li et al. [13] conducted Vickers indentations on a GaAs single crystal, yielding defects like dislocations, microtwins, stacking faults, and amorphization. Proposed amorphization mechanisms include high-pressure and shear deformation; high-pressure induced amorphization and shear deformation induced amorphization indicate the transformation from crystalline to amorphous structure. Li et al. [14] investigated cracks induced by 0.049 N load indentations in GaAs, observing shear-related crack initiation, dislocation generation, lattice distortion, and amorphous band formation. Annealing eliminated the amorphous band, revealing a crack propagation via decohesion. Huang et al. [15] studied monocrystalline GaAs deformation during nanoscratching, revealing atomic-scale lattice bending in semiconductor materials. They discussed the lattice bending mechanism and found the residual stress could be responsible for the local lattice bending. Parlinska et al. [16] explored GaAs nanoindentations and nanoscratches using different indenters. The Berkovich indentations caused convergent dislocations, twins, and slip bands, while the 60° wedge indentations led to divergent bands and median cracks. They discussed the mechanism of deformation of the crystals and found that the deformation was mainly concentrated at the front of the indenter. They similarly found by TEM experiments that the crystal deformation was mainly concentrated at the front of the indenter. Wasmer et al. [17] employed nanoindentation and scratching to study gallium arsenide. They discovered twinning during indentation and slip bands and perfect dislocations during scratching. This phenomenon was attributed to differing strain rates, higher in scratching, promoting a perfect α dislocation propagation, while slower indentation velocities enable twinning nucleation from surface inhomogeneities. Wasmer et al. [18] employed scratch tests on GaAs $\left\{001\right\}$ crystals with loads (5–100 mN) and a Berkovich indenter. They unveiled the plastic deformation stages, including dislocation cloud formation, median cracks nucleation, surface radial cracks, plastic flow, lateral cracks, and chip formation. These events exhibited a power-law dependence. Elastic recovery was approximately 15%, explained by the rheological factor X. Gao et al. [19] utilized scratching and cleavage operations to enhance GaAs cleavage planes in high-power semiconductor laser cavities. Scratching with a lower load and higher speed reduced damage, while the scratch capability index (SCI) indicated the cleavage plane quality. This approach can advance semiconductor laser chip manufacturing. They also discussed the relationship between scratch quality and load and found that the load has a significant effect on scratch quality. Yu et al. [20] employed nanoscratch tests on GaAs $\left\{100\right\}$ using an atomic force microscope (AFM) with a SiO_2_ tip. Decreasing the sliding velocity increased the scratch depth. High-resolution transmission electron microscopy (HRTEM) found no lattice damage. The material removal was attributed to dynamic interfacial bond breakage. High-speed sliding resulted in a faster GaAs surface material removal, ideal for SiO_2_ polishing without surface damage. Chen et al. [21] used molecular dynamics (MD) simulations to investigate single-crystal copper nanoscratching. They observed depth-dependent subsurface changes, differing (100) and (111) plane behaviors, and identified stack faults. It was shown that the surface integrity was not only related to the scratch depth, but the surface grain orientation also had a non-negligible effect on the surface integrity. Fan et al. [22] employed oblique nanomachining to enhance GaAs machining quality. They observed an early dislocation avalanche and a favorable plasticity during cutting under certain tip conditions, particularly oblique cutting. Gao et al. [23] employed a novel method to study anisotropic stress in GaAs. They found a lower stress along $\left\{100\right\}$ than $\left\{110\right\}$ orientations. The (011) plane displayed potential as a preferential cleavage plane with improved quality. This research enhanced the understanding of cleavage mechanisms. The study discussed the stress field of the GaAs scribing process and showed that the maximum stress was concentrated at the tip of the indenter and appeared anisotropic in different directions. Wang et al. [24] employed AFM tip-based nanoscratching to create GaAs nanochannels, studying the material removal and subsurface damage. Depths below 11 nm favored cutting over plowing, inducing stacking faults, dislocations, nanocrystallization, and amorphization. Wu et al. [25] probed GaAs surface defects using a conductive atomic force microscope (C-AFM). Scratches showed a higher edge current, influenced by the load. Etching increased currents, with scratch-induced Schottky barrier height changes. Fang et al. [26] studied Si and GaAs nanomechanical properties via nanoindentation and nanoscratch. Results showed a decreased Young’s modulus and hardness with a higher load, hold time, and cycles. GaAs exhibited a pop-in effect, and the wear behavior varied with the feed and load. The scratch technique used the material removal volume to evaluate hardness. The study found an effect of the applied load on the GaAs surface quality, which related to the surface hardness and Young’s modulus.

However, due to the high cost of experimental research and the stringent requirements for experimental environments, many scholars are gradually adopting a combination of MD simulation with experimental research. The MD simulation studies of materials are widely used to investigate the mechanical behavior and deformation mechanisms of materials at the nanoscale. It has been widely applied in the study of atomic-scale surface deformation and crystal structure and is suitable for the study of properties that are difficult to measure with many traditional experimental methods [27,28,29,30,31]. Li et al. [32] employed molecular dynamics simulations to investigate the influence of cracking on GaAs deformation in different crystal orientations during processing. Their findings revealed cracking-induced alterations in atomic-level deformation behavior, attributed to the tensile stress distribution and fracture surface variations. The anisotropy induced by the surface grain orientation, which has an important effect on the surface defects, can also be seen by MD simulation. Xu et al. [33] used molecular dynamics simulations to investigate GaAs crystal anisotropy during nanoscratching. They found significant anisotropic effects on the deformation, residual stress, and surface properties, offering new insights into the material behavior. The study also confirmed that the anisotropy of the surface grain direction had an important influence on the distribution of residual stresses. Yi et al. [34] utilized molecular dynamics to examine GaAs nanoscratching in chemical mechanical polishing. Phase transformation and amorphization were the dominant deformation mechanisms. Anisotropic effects were observed, with varied scratching resistance and friction coefficients among different GaAs crystal orientations, providing insights into the mechanical wear in GaAs polishing. The study also confirmed that the anisotropy of the surface grain direction had an important influence on the scratching forces. Chen et al. [35] employed molecular dynamics simulations to explore surface and subsurface deformations in gallium arsenide during nanocutting. Dislocations, phase transformations, and anisotropic effects were investigated, providing insights into performance-affecting factors in GaAs machining. Li et al. [36] used molecular dynamics simulations to explore plowing-induced deformation in GaAs. They observed crack initiation, propagation, and dislocation-dominated plasticity, providing atomic-level insights into a novel deformation pattern in GaAs during plowing. The MD simulations also found that the deformation and high stress areas were mainly distributed at the front end of the indenter, which was consistent with the experimentally generated phenomena. Fan et al. [37] simulated the AFM tip-based hot machining of GaAs at temperatures of 600 K, 900 K, and 1200 K, revealing reduced cutting forces, increased friction, enhanced material removal rate, and ductile response with dislocations, along with chip densification during hot cutting. Fan et al. [38] studied nanoscale friction using MD simulations and AFM nanoscratch experiments on gallium arsenide. They examined the scratch depth effects, revealing a size-dependent behavior. The study found correlations between MD simulations and AFM experiments, indicating a specific scratch energy insensitivity to the tool geometry and scratch speed. However, the pile-up and kinetic coefficient of friction were influenced by the tool’s tip geometry. Fan et al. [39] investigated a diamond wear during AFM-based nanomachining of GaAs via MD simulations. They observed the diamond tip’s elastic–plastic deformation and transformation from a cubic to graphite structure, identifying graphitization as the dominant wear mechanism, introducing a novel method for quantifying the graphitization conversion rate. Chen et al. [40] investigated gallium arsenide’s crack formation during nanocutting. They found a transition from dislocation to phase transformation at higher cutting speeds, with more cracks at greater depths. Deformation shifted from ductile to ductile–brittle, with cracks at the amorphous–single crystal boundaries. Tensile stress was concentrated at crack tips. Taper-cutting experiments revealed a 25 nm brittle–ductile transition depth, supported by transmission electron microscopy (TEM) showing microcracks and polycrystals in the subsurface, aligning with simulation findings. Li et al. [41] reviewed molecular dynamics simulations in tip-based nanomachining (TBN), covering material-specific models, TBN mechanisms, and future prospects, offering valuable insights for further research in this field. The study provided a systematic overview of the molecular dynamics study of TBN, showing that the molecular dynamics approach was applicable to the study of mechanical properties and surface defects. Fan et al. [42] used molecular dynamics to explain ductile plasticity in polycrystalline gallium arsenide during nanoscratching, emphasizing the dislocation nucleation at grain boundaries and its impact on material behavior. Rino et al. [43] studied structural phase transformation in crystalline gallium arsenide under a 22 GPa pressure, with a reverse transformation observed at 10 GPa, showing hysteresis. Molecular dynamics results matched experiments, estimating a 0.06 eV energy barrier. The simulation results showed that there was a clear relationship between the stresses and the changes in the crystal structure. Kodiyalam et al. [44] investigated pressure-induced structural transformation in gallium arsenide nanocrystals, with nucleation occurring at the surface, leading to inhomogeneous deformation and grain boundaries. It was also found that the region of high-pressure distribution had an important influence on the transformation of the crystal structure. Parasolov et al. [45] developed a nanoindentation model using molecular dynamics on GaAs. Above 100 K, nanoindentation led to increased point defects in GaAs atomic layers, attributed to thermal energy fluctuations and external stress. Gular et al. [46] conducted geometry optimization calculations on GaAs up to 25 GPa using a Stillinger–Weber potential. They determined a B3 to B1 phase transition at 17 GPa and evaluated various material properties, providing valuable insights for future GaAs pressure studies. The comprehensive analysis of MD simulations shows that anisotropy has an important effect on surface defects and crystal structure, and the MD simulation method is also applicable to the study of micromechanical properties in nanofabrication; the effect of crystal orientation will be further investigated in this study.

Existing studies have shown that in the processing of GaAs crystalline materials, the selection of the appropriate crystal orientation has an important impact on the performance of the final processed workpiece [8,9]. In this work, the machined surface of GaAs with different crystallographic orientations is modeled and the surface morphology and amorphous damage layer after nanopolishing are investigated; in addition, residual stresses as well as temperatures are analyzed in order to select a suitable crystallographic orientation for nanofabrication. This work utilizes MD simulations to investigate the processes of surface generation and subsurface damage. A nanoscale-polishing molecular dynamics model incorporating the microasperity structure of the actual processed surface is established. The surface topography, mechanical properties, and phase transition processes under $\left\{100\right\}$, $\left\{110\right\}$, and $\left\{111\right\}$ crystal orientations are analyzed, validating the influence of anisotropy on the surface morphology and subsurface crystal phase transformation extent. Furthermore, by analyzing the differences in surface pile-up after nanoscale polishing for three crystal orientations, this work also examines the impact of the surface crystal orientation on the temperature distribution and residual stress distribution during the nanoscale polishing process, which may have practical implications for nanoscale polishing processes.

## 2. Methods

### Simulation Methods

**Figure 1:**
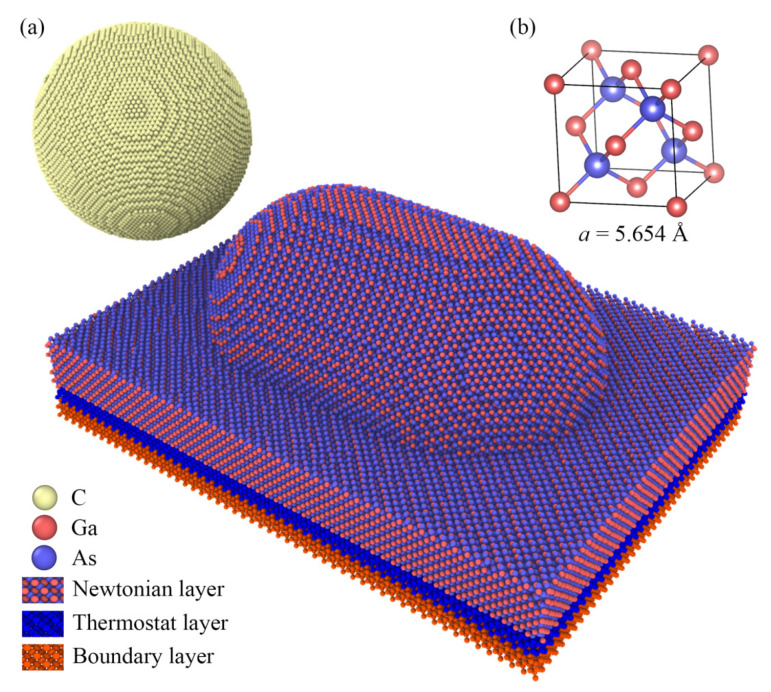
Molecular dynamics models of nanopolishing of gallium arsenide. (**a**) Model structure (**b**) GaAs crystal structure.

In comparison to the traditional nanoscale polishing model, the nanoscale polishing model employed in this study takes into account the microconvex structures present on the actual processed surface. The variables under investigation pertain to the crystallographic orientations of gallium arsenide (GaAs) surfaces during the nanoscale polishing process, specifically the $\left\{100\right\}$, $\left\{110\right\}$, and $\left\{111\right\}$ crystallographic orientations. The nanoscale polishing model for GaAs crystals, as illustrated in Figure 1, can be conceptually divided into two main components: the equivalent spherical representation of the diamond polishing tool and the GaAs surface with its microconvex structures.

As depicted in Figure 1a, the equivalent diamond polishing particle had a diameter of 12 Å, consisting of 159,486 atoms, and possessed a lattice constant of 3.57 Å. The equivalent GaAs surface was composed of two parts: a substrate with dimensions of 300 Å × 220 Å × 50 Å and microconvex structures comprising one-quarter spheres at both ends and a central half-cylinder, all with a radius of 7 Å. The centers of the spherical structures at the two ends were located at (110 Å, 110 Å, 50 Å) and (190 Å, 110 Å, 50 Å), respectively. The position of the diamond particle was (−60 Å, 110 Å, 120 Å). The total number of gallium atoms was 104,963, and the total number of arsenic atoms was 103,420. The crystallographic structure of the GaAs crystal is depicted in Figure 1b, with a lattice constant of 5.654 Å.

The equivalent model for the gallium arsenide (GaAs) surface was divided into three distinct layers, as shown in Figure 1a: the Newtonian atomic layer situated at the top, where atomic motion follows Newton’s second law and is calculated using the velocity Verlet algorithm [47]; the isothermal atomic layer in the middle, which regulates temperature changes based on the Berendsen thermostat [48]; and the fixed atomic layer at the bottom, where atomic positions and velocities are constrained to prevent atoms from escaping the boundary. In the multilayer structure, the thickness of the Newtonian layer was 100 Å (70 Å for the radius of the microconvex body and 30 Å for the basal portion), the thickness of the thermostatic layer was 10 Å, and the temperature of the boundary layer was 10 Å. In addition to the potential energy parameters, to ensure convergence, the model set boundary conditions as well as energy minimization constraints so that the model was in a steady state before nanopolishing. To enhance computational efficiency in the simulation, this work employed periodic boundary conditions for the nanoscale polishing process. Specifically, periodic boundary conditions were applied in the *y*-direction to exploit the system’s symmetric properties, while nonperiodic boundary conditions were imposed in the *x*-direction (processing direction) and the *z*-direction (normal to the surface) to ensure a realistic representation of the system.

**Figure 2:**
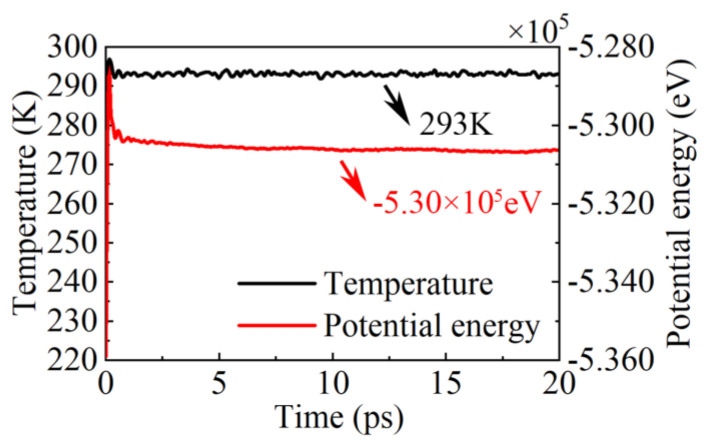
Temperature and potential energy of the relaxation process before nanopolishing.

This study utilized the Large-scale Atomic/Molecular Massively Parallel Simulator (LAMMPS) [49] for molecular dynamics simulations and employed the open visualization tool (OVITO) [50] for the visualization and postprocessing of the simulation results. The detailed parameters of the model are presented in Table 1. The simulation workflow included the prepolishing energy minimization process using the conjugate gradient method [51]. The model’s relaxation process was conducted under the NPT ensemble with a relaxation time of 100 ps. During this process, the model’s temperature gradually stabilized at room temperature (293 K) using the Nose–Hoover thermostat, and the potential energy converged to −5.30 × $10^{-5}$ eV. The temperature and potential energy changes during the relaxation process are illustrated in Figure 2. Following the relaxation of the model, the ensemble was switched to NVE, and the simulation of nanoscale polishing was performed. During the relaxation phase of the model, the temperature gradually stabilized at 293 K, and the total potential energy of the model gradually stabilized at −5.30 × $10^{-5}$ eV. In this process, the polishing speed of diamond abrasive particles was set at 100 m/s in the (0,1,0) direction, with a polishing distance of 30 nm. Before the calculations for stresses, RDF, and temperature and after the nanopolishing simulation, the model was subjected to a relaxation process, which resulted in a more stable surface structure after processing. To observe the stable structure of the surface after the nanoscale polishing process, a second relaxation process was conducted for the model, also with a relaxation time of 100 ps.

During the process of nanoscale machining, the selection of the interatomic potential energy is of paramount importance. In the case of polishing gallium arsenide (GaAs) workpieces, the interatomic potential energy functions in Ga-Ga, Ga-As, and As-As atoms are described by the Tersoff potential [52] and the parameters refers to [53]. The expression of the Tersoff potential function is shown in Equation (1). For the interatomic potential energy function in carbon–carbon (C-C) atoms in diamond polishing particles, the Tersoff potential was employed. The interatomic potential energy functions between carbon (C) atoms in diamond polishing particles and gallium (Ga) or arsenic (As) atoms in GaAs workpieces are governed by the Ziegler–Biersack–Littmark universal screening function (ZBL) potential [54]. The expression of the ZBL potential is presented in Equation (2), where the parameter inner is the distance where the switching function begins, and outer is the global cutoff for the ZBL interaction. The parameters inner of Ga-C and As-C are 31.0 and 33.0, respectively. The parameters outer of Ga-C and As-C are 12.0.

(1) $$\left\{\begin{array}{c} E=\frac{1}{2}\sum_{i}\sum_{i\neq j}V_{ij}\\ V_{ij}=f_{c}\left(r_{\mathrm{ij}}\right)\left[f_{R}\left(r_{\mathrm{ij}}\right)+b_{ij}f_{A}\left(r_{\mathrm{ij}}\right)\right] \end{array}\right.$$

where $V_{ij}$ is the Tersoff potential energy, $f_{R}$ means the two-body term, $f_{A}$ means the three-body term, $f_{C}$ means the cutoff of the coefficient.

(2) $$V_{\mathrm{ij}}=\frac{1}{4\pi \varepsilon_{0}}\frac{Z_{1}Z_{2}e^{2}}{r_{\mathrm{ij}}}\phi \left(r_{\mathrm{ij}}/a\right)$$

where $Z_{1}$, $Z_{1}$ are the number of protons in the nucleus, *e* is the electron charge, $\varepsilon_{0}$ is the permittivity of vacuum, and $\phi (r_{\mathrm{ij}}/a)$ is the universal screening function of ZBL potential.

When evaluating surface residual stresses in polished gallium arsenide (GaAs) workpieces, the von Mises stress was calculated. It was determined based on the atomic stress tensor, taking into account the combined effects of six stress components, as expressed in Equation (3). When considering temperature variations during the nanoscale polishing process, the temperature change was represented using the average kinetic energy expression [48], as shown in Equation (4).

(3) $$\sigma_{\mathrm{vm}}\left(i\right)={\left\{\frac{1}{2}\left[\begin{array}{c} {\left(\sigma_{xx}\left(i\right)-\sigma_{yy}\left(i\right)\right)}^{2}+{\left(\sigma_{yy}\left(i\right)-\sigma_{zz}\left(i\right)\right)}^{2}\\ +{\left(\sigma_{zz}\left(i\right)-\sigma_{xx}\left(i\right)\right)}^{2}+6\left(\sigma_{xy}^{2}\left(i\right)+\sigma_{yz}^{2}\left(i\right)+\sigma_{zx}^{2}\left(i\right)\right) \end{array}\right]\right\}}^{1/2}$$

where $\sigma_{\mathrm{vm}}\left(i\right)$ denotes the von Mises stress, and σ(i) denotes an atomic stress tensor.

(4) $$E_{k}=\left(3/2\right)kT$$

where $E_{k}$ represents the average atomic kinetic energy, *k* denotes the Boltzmann constant which is $1.381\times 10^{-23}\;J/K$, and *T* denotes the temperature.

## 3. Results and Discussion

### 3.1. Surface Quality

**Figure 3:**
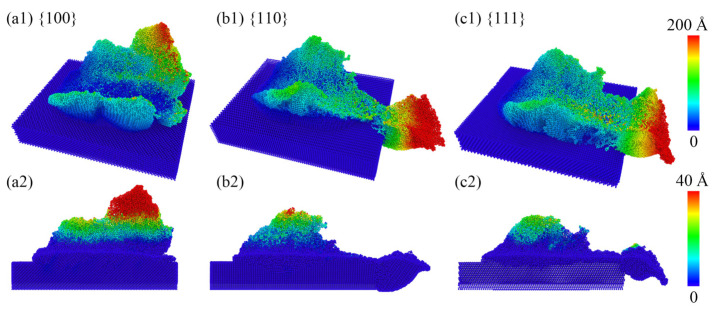
The total atomic displacement of the surface following nanoscale polishing. In panels (**a1**–**c1**), the cumulative atomic displacement is depicted, while panels (**a2**–**c2**) specifically represent atomic displacement in the *z*-axis direction.

After nanoscale polishing on the gallium arsenide (GaAs) surface, significant atomic displacements were observed primarily due to the intense interaction between diamond polishing particles and the rapidly moving surface. The atomic displacements of the three crystallographic orientations were color-coded based on the total atomic displacement and displacement along the *z*-axis, as depicted in Figure 3(a1–c1). The regions of maximum atomic displacement for different crystallographic orientations were concentrated at the ends of microprotuberances formed as chip piles, with varying specific distributions. The $\left\{100\right\}$ crystallographic orientation exhibited a maximum displacement concentration at the top of the polished chip pile, while the $\left\{110\right\}$, and $\left\{111\right\}$ orientations, as a result of their detachment from the surface after microprotuberance polishing, showed an atomic displacement accumulation along the *x*-axis. A profile analysis of atomic displacements within the chip along the *y*-axis is shown in Figure 3(a2–c2). Along the *z*-axis, atomic displacements within the residual pile-up on the surface after polishing gradually decreased for the $\left\{100\right\}$, $\left\{110\right\}$, and $\left\{111\right\}$ orientations. Due to the anisotropy caused by the crystallographic orientations, atoms from the $\left\{110\right\}$ and $\left\{111\right\}$ orientations exhibited the highest atomic displacements as they detached from the surface following their interaction with microprotuberances and diamond polishing particles, resulting in relatively smaller atomic displacements within the remaining portion compared to the $\left\{100\right\}$ orientation.

**Figure 4:**
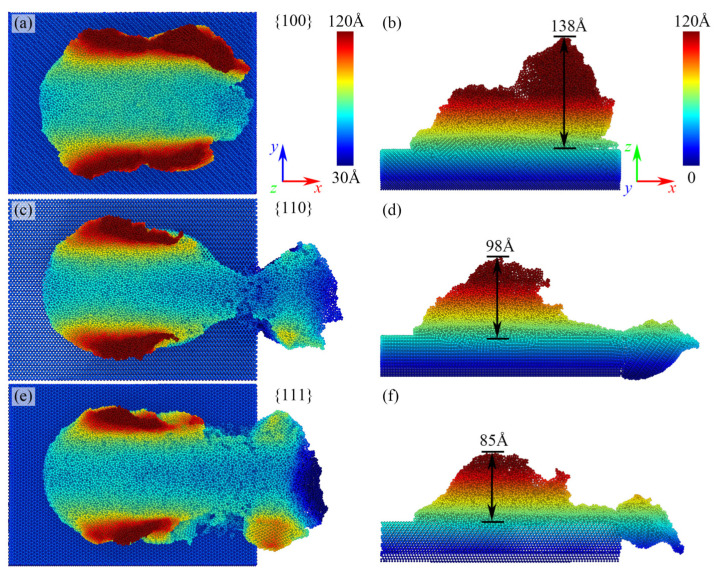
The surface quality (**a**,**c**,**e**) in the *z*-direction view, and the cross-sectional view (**b**,**d**,**f**) in the *y*-direction after nanoscale polishing.

After analyzing the atomic displacements following nanoscale polishing, postprocessing was carried out based on the *z*-coordinate positions of gallium arsenide polished surface atoms. As illustrated in Figure 4, observations from the *z*-direction top view and *z*-direction cross-sectional view revealed significant alterations in atomic distribution after nanoscale polishing. Notably, different crystallographic orientations of microprotrusions exhibited distinct impacts on the surface quality following polishing. Specifically, microprotrusions with a $\left\{100\right\}$ crystallographic orientation did not disintegrate along with the diamond polishing particles during nanoscale polishing; instead, they accumulated on the preexisting surface, resulting in a maximum surface asperity height of 138 Å. In contrast, atoms from the $\left\{110\right\}$ and $\left\{111\right\}$ crystallographic orientations split into two parts after nanoscale polishing, with pile-up heights of 98 Å and 85 Å, respectively, exerting a lesser influence on the postpolishing surface quality.

### 3.2. Mechanical Property

**Figure 5:**
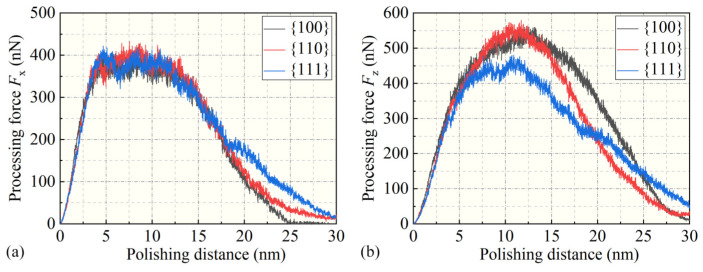
The relationship between (**a**) the tangential force, $F_{x}$, and (**b**) the normal force, $F_{z}$, with respect to the polishing distance.

In order to further investigate the influence of surface crystallographic anisotropy on mechanical properties, the normal force $F_{z}$ and tangential force $F_{x}$ during nanoscale polishing were separately calculated, as shown in Figure 5. The relationships between the normal force $F_{z}$ and the polishing distance, as well as the tangential force $F_{x}$ and the polishing distance, were analyzed. After the contact between diamond polishing particles and gallium arsenide microprotrusions, the contact force gradually increased with the moving distance, reaching a maximum value before gradually decreasing. Furthermore, it could be observed that $F_{z}$ was numerically greater than $F_{x}$. For polishing distances less than 5 nm, there were no significant differences in $F_{z}$ and $F_{x}$ among the three crystallographic orientations. However, when the polishing distance was between 5 nm and 20 nm, significant differences in $F_{z}$ and $F_{x}$ appeared among the three crystallographic orientations, and after the diamond polishing particles left the microprotrusions, both $F_{z}$ and $F_{x}$ gradually decreased to their minimum values.

**Figure 6:**
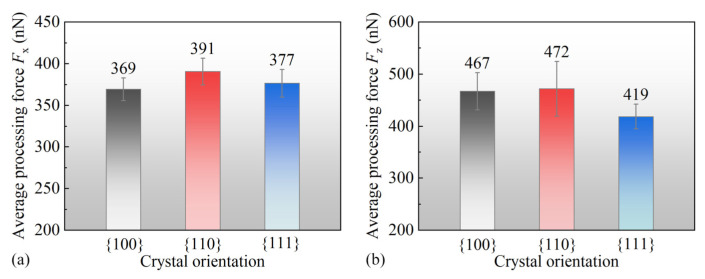
The mean contact force within a range of 5 nm to 20 nm in nanoscale polishing. (**a**) $F_{x}$; (**b**) $F_{z}$.

After calculating the contact forces in three crystallographic orientations, the average contact forces within the nanoscale polishing range of 5–20 nm were determined, as illustrated in Figure 6. It is evident that the $F_{z}$ and $F_{x}$ components were highest for the $\left\{110\right\}$ crystallographic orientation. Conversely, the $F_{x}$ component was at its minimum for the $\left\{100\right\}$ orientation, indicating reduced interatomic interactions. This observation aligned with the earlier analysis of the surface atomic displacement. It is plausible that atomic displacements in the *x*-direction were limited due to the nanopolishing, resulting in a lesser interaction for $\left\{100\right\}$. In contrast, the $\left\{110\right\}$ and $\left\{111\right\}$ orientations exhibited larger $F_{x}$ values, implying stronger interatomic interactions, potentially leading to the detachment of some atoms as the microprotrusions decomposed due to the enhanced $F_{x}$. In terms of $F_{z}$, the $\left\{111\right\}$ orientation experienced the lowest contact force, suggesting weaker interactions in the *z*-direction. This may be correlated with the earlier observation of the lowest atomic stacking height in the *z*-direction. A further investigation is required to understand its implications on the subsurface atomic structure.

### 3.3. Amorphization Analysis

**Figure 7:**
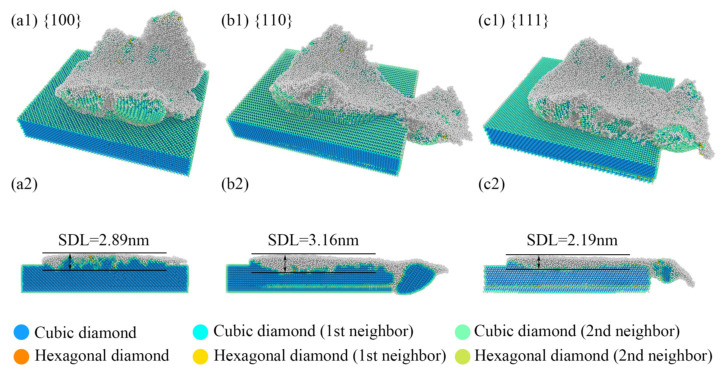
The thickness of the subsurface damage layer in the (**a1**,**b1**,**c1**) *z*-direction view, and (**a2**,**b2**,**c2**) *y*-side cross-sectional image after nanoscale polishing.

After nanoscale polishing, the anisotropy not only influences the mechanical properties but also results in differences in the crystal structure of the subsurface after processing. By examining the crystal structures of subsurface atoms, as depicted in Figure 7, it could be observed that following nanoscale polishing, most of the remaining atoms on the processed surface underwent an amorphization process, with only the outermost atoms of the microconvex polished surface retaining their original structure, namely the cubic diamond structure. From the cross-sectional view on the *y*-*z* plane, it is evident that the thickness of the subsurface damaged layer (SDL) varied after nanoscale polishing, with the $\left\{110\right\}$ crystal orientation reaching a maximum thickness of 3.16 nm, the $\left\{100\right\}$ crystal orientation measuring 2.89 nm, and the $\left\{111\right\}$ crystal orientation having a minimum thickness of 2.19 nm. Consistent with the previous analysis of mechanical properties, the thickness of the SDL exhibited a similar trend to the variation in $F_{z}$ values, potentially attributed to the varying degrees of *z*-directional interactions resulting from the crystal anisotropy.

**Figure 8:**
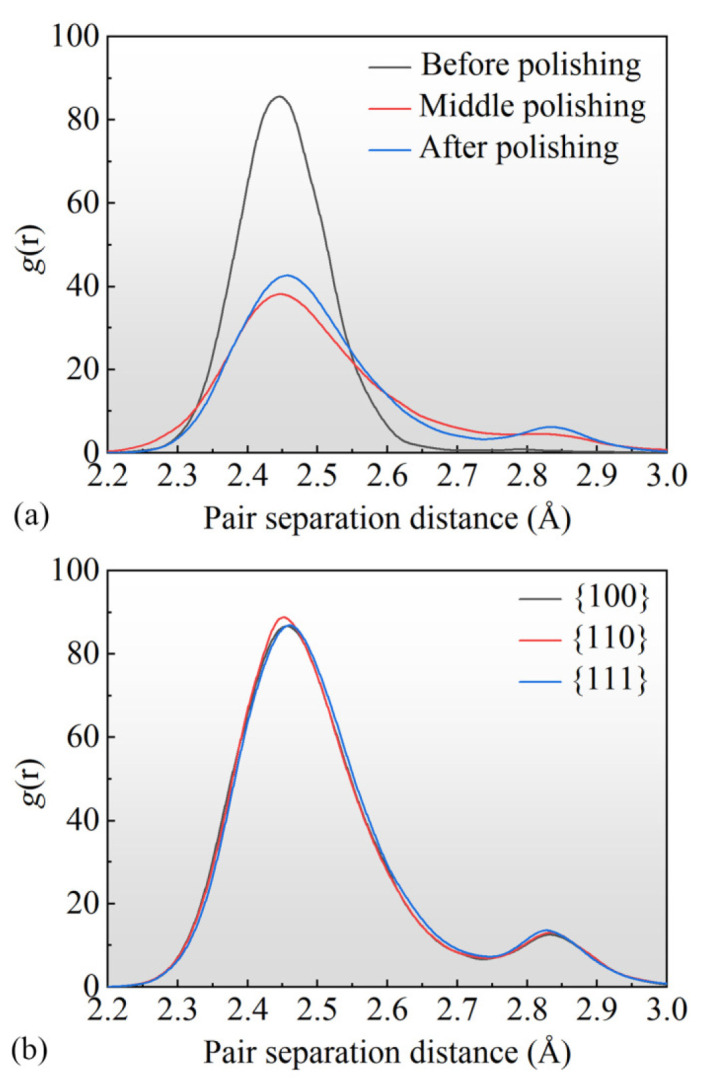
The RDF curve for the nanopolishing process. (**a**) Different stages of the machining process, (**b**) different crystal orientations.

The thickness of the amorphous SDL layer exhibited anisotropy, indicating that the selection of different crystallographic orientations for processing had an influence on the crystal structure within the polished surface. In order to further investigate the subsurface damage process, an analysis was conducted using the radial distribution function (RDF), as shown in Figure 8. The RDF is a commonly used function for studying crystal structures, representing the distribution of atomic distances, with different peaks in the curve characterizing different crystal structures. As depicted in Figure 8a, RDF calculations were performed for the processed surface before, during, and after polishing. It can be observed that during the nanoscale polishing process, there was a decrease in the peak at 2.45 Å and an increase in the peak at 2.85 Å, indicating that some crystal structures with an atomic spacing of 2.45 Å were disrupted during the processing and transformed into structures with an atomic spacing of 2.85 Å, and this process was irreversible. For different crystal orientations, as shown in Figure 8b, it can be seen that the $\left\{110\right\}$ orientation exhibited a higher peak at 2.85 Å compared to other orientations, indicating a lower proportion of amorphization atoms in the residual atoms after processing for the $\left\{110\right\}$ orientation.

### 3.4. Analysis of Temperature Distribution

**Figure 9:**
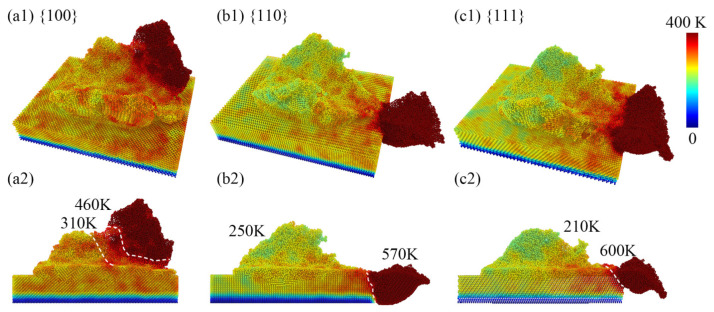
The temperature distribution of the surface quality after nanoscale polishing (**a1**–**c1**), and the lateral cross-sectional perspective (**a2**–**c2**).

Figure 9(a1–c1) represents the temperature distribution on the surface during the nanoscale polishing process. It can be observed that atoms with higher total displacements correspond to regions with higher temperatures, consistent with the previous analysis of atomic displacements. This observation indicates a relationship between the temperature distribution and the total atomic displacement during nanoscale polishing. Figure 9(a2–c2) displays a side view of the temperature distribution along the *y*-axis. It is evident that the temperature of $\left\{111\right\}$ crystal facet debris reached a maximum of 600 K, while the surface atom temperature reached a minimum of 210 K. In contrast, the $\left\{100\right\}$ facet debris exhibited a minimum temperature of 460 K, with the surface temperature reaching a maximum of 310 K. The opposite trends in temperature variation between different crystal facets during nanoscale grinding may be attributed to the fact that debris atoms carry away more heat, resulting in lower residual atomic temperatures on the surface.

**Figure 10:**
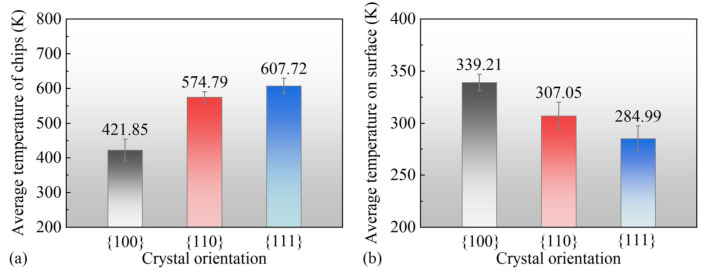
The average temperature profiles subsequent to nanoscale polishing, with distinct delineations for (**a**) the chip-removal atomic domain and (**b**) the residual-surface atomic domain.

In Figure 10, the average temperatures and errors in different regions after nanopolishing with different crystal orientations are presented, which are consistent with the trends in Figure 10. The average temperature of the $\left\{100\right\}$ crystal orientation for the cutting chips was the lowest at 421.85 K, while the average temperature of surface atoms was the highest at 339.21 K. On the other hand, the $\left\{111\right\}$ crystal orientation exhibited the highest average temperature for the cutting chips at 607.725 K, with the surface atoms having an average temperature of 284.99 K. From the statistical analysis of the average temperatures, it could be inferred that following nanopolishing, the cutting chip atoms in the $\left\{100\right\}$ crystal orientation did not separate into two parts with the movement of the diamond polishing particles. This resulted in a higher temperature accumulation, indirectly providing a higher temperature environment for amorphization, possibly leading to a more pronounced degree of amorphization. In contrast, the $\left\{111\right\}$ crystal orientation showed a lower average temperature for the remaining surface atoms after nanopolishing, which may have resulted in a lower degree of amorphization.

### 3.5. Analysis of Residual Stress

**Figure 11:**
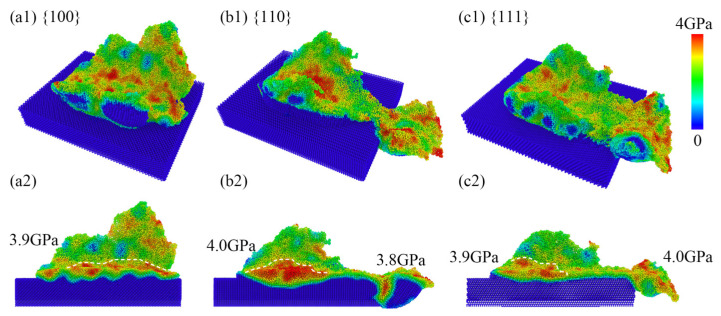
The stress distribution in the (**a1**–**c1**) region and the cross-sectional view on the (**a2**–**c2**) *y*-plane after nanoscale polishing.

The degree of crystal amorphization is not only related to the temperature environment but also to the internal stress intensity within the region. In Figure 11, the visualization results of the von Mises stress distribution on the surface after nanoscale polishing are presented. It can be observed in Figure 11(a1–c1) that following nanoscale polishing, there existed a high-stress distribution region (in red) with values ranging from 3 to 4 GPa on the surface in contact with the polishing particles, as well as stress distribution regions of 1–2 GPa, corresponding to the previously mentioned amorphous surface atomic regions. In the y-directional cross-sectional view, it can be observed that the highest stress in the SDL layer on the surface after three crystallographic directions processing was approximately 3.9 GPa. The stress distribution in the $\left\{110\right\}$ and $\left\{111\right\}$ facets was generally similar to that on the surface, although specific numerical values require further calculation.

**Figure 12:**
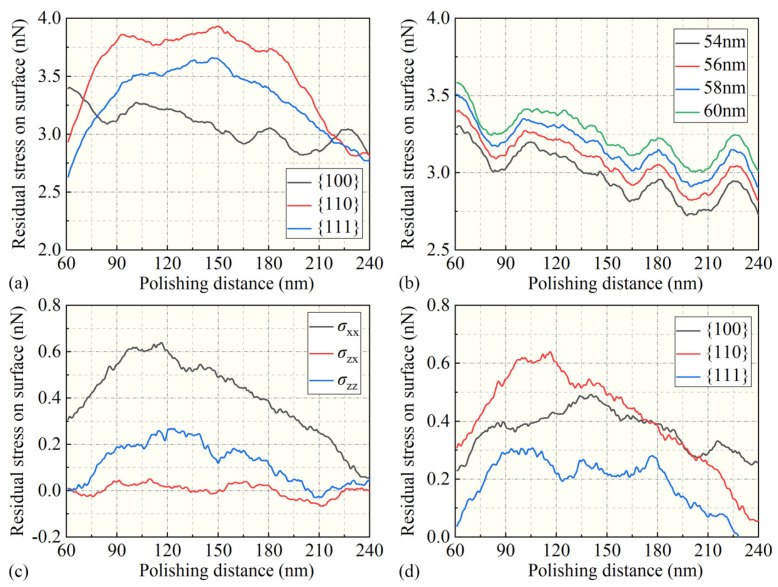
(**a**) The distribution of total stresses along different crystal orientations, (**b**) the relationship between residual stresses and depth distribution, (**c**) the stress components along different directions, and (**d**) the residual stress components along different crystal orientations in the processing direction, $\sigma_{\mathrm{xx}}$, after nanoscale polishing.

As shown in Figure 11, for the calculation of residual stresses on the processed surface, Figure 12a illustrates the distribution of total residual stresses at a depth of 56 nm along the *z*-axis for different crystallographic orientations. It can be observed that the 110 orientation exhibited the highest stress, followed by the $\left\{111\right\}$ orientation, while the $\left\{100\right\}$ orientation showed the lowest residual stress. The residual stress distribution at various depths along the $\left\{100\right\}$ orientation is depicted in Figure 12b, revealing a generally negative correlation between residual stress and depth. The minimum residual stress was observed at a depth of 54 nm, while stress levels increased closer to the surface, reaching higher values at a depth of 60 nm. Furthermore, an analysis of different stress components on the $\left\{100\right\}$ orientation is presented in Figure 12c. It is evident that the shear stress component $\sigma_{\mathrm{xx}}$ in the xx direction was significantly larger than in other directions. Additionally, a comparison of the xx direction stress component $\sigma_{\mathrm{xx}}$ was made among different crystallographic orientations. Notably, the $\left\{111\right\}$ orientation exhibited a smaller stress component value, whereas the $\left\{110\right\}$ orientation displayed the largest residual stress component value. These findings may have implications for the stability of the crystal structure and surface quality following surface polishing.

## 4. Conclusions

In this work, MD simulations were employed to investigate the processes of surface generation and subsurface damage. A nanoscale polishing molecular dynamics model was established, taking into consideration the microconvex structures of the actual processed surface. The analysis encompassed surface morphology, mechanical properties, and amorphization processes under the $\left\{100\right\}$, $\left\{110\right\}$, and $\left\{111\right\}$ crystal orientations, thus confirming the influence of anisotropy on surface morphology and subsurface crystalline amorphization extent. After analyzing the disparities in surface pile-up following nanoscale polishing in three crystal orientations, it was discerned that the $\left\{111\right\}$ crystal orientation exhibited a lower residual atomic height and a lower normal contact force during the processing. Additionally, an investigation of subsurface crystalline amorphization revealed a thinner amorphous layer beneath the $\left\{111\right\}$ crystal orientation. In the RDF analysis, it was observed that the proportion of atoms undergoing amorphization was slightly lower under the $\left\{110\right\}$ crystal orientation compared to the other two orientations. Furthermore, this work examined the influence of the surface crystal orientation on the temperature distribution and residual stress distribution during the nanoscale polishing process. Regarding temperature, the $\left\{111\right\}$ crystal orientation exhibited lower surface temperatures during the processing. In terms of stress, it was found that the tangential residual stress component, $\sigma_{\mathrm{xx}}$, was larger compared to the normal residual stress component, $\sigma_{\mathrm{zz}}$. Additionally, $\sigma_{\mathrm{xx}}$ under the $\left\{111\right\}$ crystal orientation was lower. Considering the comprehensive analysis of postpolishing surface morphology, contact forces, SDL thickness, temperature, and stress distribution, it can be concluded that the microconvex structures under the $\left\{111\right\}$ crystal orientation have a lesser impact on surface quality and subsurface amorphization after polishing, which may hold significance for practical nanoscale polishing processes.

## Figures and Tables

**Table 1:** The MD simulation parameters.

Simulation Parameters	Value
Material of the workpiece	Gallium arsenide (GaAs)
Material of the nanopolishing grit	Diamond (C)
Dimension of the workpiece (nm)	30×22×5
Radius of the nanopolishing grit (nm)	6
Surface crystal orientation of the workpiece	$\left\{100\right\}$, $\left\{110\right\}$, and $\left\{111\right\}$
Potential function	Tersoff, ZBL
Nanogrinding speed (m/s)	100
Ambient temperature (K)	300
Nanogrinding distance (nm)	30
Timestep (fs)	1

## Data Availability

The original contributions presented in the study are included in the article, further inquiries can be directed to the corresponding author.

## References

[1] Ahn H.J., Chang W.I., Kim S.M., Park B.J., Yook J.M., Eo Y.S. (2019). 28 GHz GaAs pHEMT MMICs and RF front-end module for 5G communication systems. Microw. Opt. Technol. Lett..

[2] Chow T.P., Omura I., Higashiwaki M., Kawarada H., Pala V. (2017). Smart power devices and ICs using GaAs and wide and extreme bandgap semiconductors. IEEE Trans. Electron Devices.

[3] Hayati-Roodbari N., Wheeldon A., Hendler C., Fian A., Trattnig R. (2021). Ohmic contact formation for inkjet-printed nanoparticle copper inks on highly doped GaAs. Nanotechnology.

[4] Gao J., Zhou H., Du J., Peng W., Lin Y., Xiao C., Yu B., Qian L. (2022). Effect of counter-surface chemical activity on mechanochemical removal of GaAs surface. Tribol. Int..

[5] Dinodiya S., Bhargava A. (2022). A comparative analysis of pressure sensing parameters for two dimensional photonic crystal sensors based on Si and GaAs. Silicon.

[6] Hao D., Zhang W., Liu X., Liu Y. (2021). A low insertion loss variation trombone true time delay in GaAs pHEMT monolithic microwave integrated circuit. IEEE Microw. Wirel. Components Lett..

[7] Jordan A., Von Neida A., Caruso R. (1986). The theoretical and experimental fundamentals of decreasing dislocations in melt grown GaAs and InP. J. Cryst. Growth.

[8] Xu M., Wu Y., Koybasi O., Shen T., Ye P. (2009). Metal-oxide-semiconductor field-effect transistors on GaAs (111) A surface with atomic-layer-deposited Al_2_O_3_ as gate dielectrics. Appl. Phys. Lett..

[9] Bisson S., Kulp T., Levi O., Harris J., Fejer M. (2006). Long-wave IR chemical sensing based on difference frequency generation in orientation-patterned GaAs. Appl. Phys. B.

[10] Johnson K.L., Johnson K.L. (1987). Contact Mechanics.

[11] Hill R. (1998). The Mathematical Theory of Plasticity.

[12] Gao R., Jiang C., Walker D., Li H., Zheng Z. (2022). Molecular dynamics study on mechanical cleavage mechanisms of GaAs and experimental verification. Ceram. Int..

[13] Li Z., Liu L., Wu X., He L., Xu Y. (2002). Indentation induced amorphization in gallium arsenide. Mater. Sci. Eng. A.

[14] Li Z., Liu L., He L., Xu Y., Wu X. (2001). Shear-activated indentation crack in GaAs single crystal. J. Mater. Res..

[15] Wu Y., Huang H., Zou J. (2012). Lattice bending in monocrystalline GaAs induced by nanoscratching. Mater. Lett..

[16] Parlinska-Wojtan M., Wasmer K., Tharian J., Michler J. (2008). Microstructural comparison of material damage in GaAs caused by Berkovich and wedge nanoindentation and nanoscratching. Scr. Mater..

[17] Wasmer K., Parlinska-Wojtan M., Gassilloud R., Pouvreau C., Tharian J., Micher J. (2007). Plastic deformation modes of gallium arsenide in nanoindentation and nanoscratching. Appl. Phys. Lett..

[18] Wasmer K., Parlinska-Wojtan M., Graça S., Michler J. (2013). Sequence of deformation and cracking behaviours of Gallium–Arsenide during nano-scratching. Mater. Chem. Phys..

[19] Gao R., Jiang C., Lang X., Dong K., Li F. (2021). Experimental investigation of influence of scratch features on GaAs cleavage plane during cleavage processing using a scratching capability index. Int. J. Precis. Eng. Manuf.-Green Technol..

[20] Yu B., Gao J., Chen L., Qian L. (2015). Effect of sliding velocity on tribochemical removal of gallium arsenide surface. Wear.

[21] Chen J., Liang Y., Chen M., Bai Q., Tang Y. (2009). A study of the subsurface damaged layers in nanoscratching. Int. J. Abras. Technol..

[22] Fan P., Katiyar N.K., Goel S., He Y., Geng Y., Yan Y., Mao H., Luo X. (2023). Oblique nanomachining of gallium arsenide explained using AFM experiments and MD simulations. J. Manuf. Process..

[23] Gao R., Jiang C., Lang X., Zheng Z., Jiang J., Huang P. (2022). Study on mechanical cleavage mechanism of GaAs via anisotropic stress field and experiments. IEEE Trans. Semicond. Manuf..

[24] Wang J., Yan Y., Jia B., Geng Y. (2021). Study on the processing outcomes of the atomic force microscopy tip-based nanoscratching on GaAs. J. Manuf. Process..

[25] Wu L., Yu B., Fan Z., Zhang P., Feng C., Chen P., Ji J., Qian L. (2020). Effects of normal load and etching time on current evolution of scratched GaAs surface during selective etching. Mater. Sci. Semicond. Process..

[26] Fang T.H., Chang W.J., Lin C.M. (2005). Nanoindentation and nanoscratch characteristics of Si and GaAs. Microelectron. Eng..

[27] Xuan T., Li J., Li B., Fan W. (2021). Effects of the non-uniform magnetic field on the shear stress and the microstructure of magnetorheological fluid. J. Magn. Magn. Mater..

[28] Mahmood A., Chen S., Chen C., Weng D., Wang J. (2019). Molecular dynamics study of temperature influence on directional motion of gold nanoparticle on nanocone surface. J. Phys. Chem. C.

[29] Li B. (2020). Molecular Dynamics Simulations of Deformation Behavior of AlN in Nanoscratching. Proceedings of the International Manufacturing Science and Engineering Conference.

[30] Yin Z., Zhu P., Li B., Xu Y., Li R. (2021). Atomic simulations of deformation mechanism of 3C-SiC polishing process with a rolling abrasive. Tribol. Lett..

[31] Chen W., Wang W., Liang H., Zhu P. (2021). Molecular dynamics simulations of lubricant outflow in porous polyimide retainers of bearings. Langmuir.

[32] Li B., Li J., Xu J., Xuan T., Fan W. (2023). Effects of cracking on the deformation anisotropy of GaAs with different crystal orientations during scratching using molecular dynamics simulations. Tribol. Int..

[33] Xu X., Fan W., Li B., Cao J. (2021). Influence of GaAs crystal anisotropy on deformation behavior and residual stress distribution of nanoscratching. Appl. Phys. A.

[34] Yi D., Li J., Zhu P. (2018). Study of nanoscratching process of GaAs using molecular dynamics. Crystals.

[35] Chen C., Lai M., Fang F. (2021). Subsurface deformation mechanism in nano-cutting of gallium arsenide using molecular dynamics simulation. Nanoscale Res. Lett..

[36] Li B., Li J., Fan W., Xuan T., Xu J. (2022). The dislocation-and cracking-mediated deformation of single asperity GaAs during plowing using molecular dynamics simulation. Micromachines.

[37] Fan P., Goel S., Luo X., Yan Y., Geng Y., He Y., Wang Y. (2021). Molecular dynamics simulation of AFM tip-based hot scratching of nanocrystalline GaAs. Mater. Sci. Semicond. Process..

[38] Fan P., Goel S., Luo X., Upadhyaya H.M. (2021). Atomic-scale friction studies on single-crystal gallium arsenide using atomic force microscope and molecular dynamics simulation. Nanomanuf. Metrol..

[39] Fan P., Goel S., Luo X., Yan Y., Geng Y., Wang Y. (2021). An atomistic investigation on the wear of diamond during atomic force microscope tip-based nanomachining of gallium arsenide. Comput. Mater. Sci..

[40] Chen C., Lai M., Fang F. (2021). Study on the crack formation mechanism in nano-cutting of gallium arsenide. Appl. Surf. Sci..

[41] Li Z., Yan Y., Wang J., Geng Y. (2020). Molecular dynamics study on tip-based nanomachining: A review. Nanoscale Res. Lett..

[42] Fan P., Goel S., Luo X., Yan Y., Geng Y., He Y. (2021). Origins of ductile plasticity in a polycrystalline gallium arsenide during scratching: MD simulation study. Appl. Surf. Sci..

[43] Rino J.P., Chatterjee A., Ebbsjö I., Kalia R.K., Nakano A., Shimojo F., Vashishta P. (2002). Pressure-induced structural transformation in GaAs: A molecular-dynamics study. Phys. Rev. B.

[44] Kodiyalam S., Kalia R.K., Kikuchi H., Nakano A., Shimojo F., Vashishta P. (2001). Grain boundaries in gallium arsenide nanocrystals under pressure: A parallel molecular-dynamics study. Phys. Rev. Lett..

[45] Prasolov N., Brunkov P., Gutkin A. (2017). Molecular dynamics simulations of GaAs-crystal surface modifications during nanoindentation with AFM tip. J. Phys. Conf. Ser..

[46] Güler E., Güler M. (2014). Phase transition and elasticity of gallium arsenide under pressure. Mater. Res..

[47] Spreiter Q., Walter M. (1999). Classical molecular dynamics simulation with the Velocity Verlet algorithm at strong external magnetic fields. J. Comput. Phys..

[48] Berendsen H.J., Postma J.P.M., van Gunsteren W.F., DiNola A., Haak J.R. (1984). Molecular dynamics with coupling to an external bath. J. Chem. Phys..

[49] Van Gunsteren W., Berendsen H.J. (1977). Algorithms for macromolecular dynamics and constraint dynamics. Mol. Phys..

[50] Stukowski A. (2009). Visualization and analysis of atomistic simulation data with OVITO–the Open Visualization Tool. Model. Simul. Mater. Sci. Eng..

[51] Štich I., Car R., Parrinello M., Baroni S. (1989). Conjugate gradient minimization of the energy functional: A new method for electronic structure calculation. Phys. Rev. B.

[52] Erhart P., Albe K. (2005). Analytical potential for atomistic simulations of silicon, carbon, and silicon carbide. Phys. Rev. B.

[53] Albe K., Nordlund K., Nord J., Kuronen A. (2002). Modeling of compound semiconductors: Analytical bond-order potential for Ga, As, and GaAs. Phys. Rev. B.

[54] Ziegler J., Biersack J., Littmark U. (1985). The Stopping and Range of Ions in Solids.

